# Difficulté de la prise en charge de la Leucémie aiguë au cours de la grossesse au Maroc

**Published:** 2012-09-05

**Authors:** Rajaa Tissir, Mouna Lamchahab, Mustapha Benhassou, Meryeme Quachouh, Mohammed Rachid, Said Benchakroun, Asmaa Quessar

**Affiliations:** 1 Service d'hématologie et d'Oncologie pédiatrique Hôpital 20 août 1953, Faculté de Médecine et de Pharmacie, Université Ain Chok, Casablanca, Maroc; 2 Service de gynécologie et obstétrique, CHU Ibn Rochd Casablanca Maroc

**Keywords:** Leucémie aiguë, grossesse, chimiothérapie, complication de la grossesse, fœtus, Acute leukemia, chemotherapy, pregnancy, complication of pregnancy, fetus

## Abstract

L'association de la leucémie aiguë (LA) et grossesse est rare. Son incidence est estimée à 1/100 000 grossesses. Dans les 2/3 des cas ce sont des leucémies aiguës myéloblastiques. Le diagnostic est généralement fait pendant le 2ème et le 3ème trimestre. Elle pose un problème éthique et thérapeutique car La chimiothérapie au cours de la grossesse expose le fœtus aux complications. Tout retard ou changement de traitement pour sauvetage fœtal risque d'aggraver le pronostic maternel. L'objectif de ce travail est de décrire les particularités de la prise en charge de la leucémie aiguë chez la femme enceinte. Etude prospective descriptive des femmes enceintes atteintes de leucémie aiguë colligées au service d'hématologie et d'oncologie pédiatrique à l'hôpital 20 Août du CHU IBN ROCHD de Casablanca depuis janvier 2009 à Août 2011. Le diagnostic de LA est fait selon les critères de l'OMS. Le traitement était instauré après consentement des patientes selon le type de LA. Huit cas de leucémies aiguës au cours de la grossesse sont colligés au service d'hématologie et d'oncologie pédiatrique à l'hôpital 20 Août du CHU IBN ROCHD de Casablanca. Sept leucémies aiguës myéloblastiques et une leucémie aiguë lymphoblastique. Quatre cas ont été diagnostiqués durant le 1er trimestre de la grossesse, Un cas pendant le 2ème trimestre et 3 cas pendant le 3ème trimestre. L'accouchement est par césarienne programmée dans 2 cas et par voie basse dans 3 cas dont un est prématuré, 2 fausses couches et une mort fœtal après décès maternel. Trois patientes ont reçue la chimiothérapie durant la grossesse après consentement éclairé et les 5 autres après l'accouchement. Les 5 nouveau-nés sont bien portants avec un bilan malformatif négatif. L'association de la leucémie aiguë et grossesse est un évènement rare. Elle nécessite une prise en charge multidisciplinaire tenant compte des impératifs de la maladie et de son traitement, de la femme et de son désire de grossesse ainsi que des dimensions éthiques et morales.

## Introduction

L'association leucémie aiguë (LA) et grossesse est rare, son incidence est estimée à 1 /75 000 – 100 000 grossesses [1, 2]. Plus de 350 cas ont été rapportés dans la littérature [2–4]. La plus part des antimitotiques traversent le placenta, du fait de leurs faible poids moléculaire et de la solubilité élevée affectant le devenir fœtal à court et à long terme [5, 6]. Elle soulève de nombreux problèmes: diagnostic, thérapeutique. La LA au cours de la grossesse met en jeu le pronostic fœtal et maternel rendant le diagnostic et le traitement difficile. L'objectif de ce travail est de décrire les particularités de la prise en charge de la LA au cours de la grossesse.

## Méthodes

C'est une étude prospective descriptive menée au service d'hématologie et d'oncologie pédiatrique à l'hôpital 20 août du CHU IBN ROCHD de Casablanca depuis janvier 2009 à Août 2011. Toutes patientes enceintes atteintes de LA au cours de la grossesse a été incluse. Les grossesses survenues après le traitement ont été exclues. Le diagnostic de LA est fait selon les critères de l'OMS: morphologiques, immuno-phénotypiques et cytogénétiques. Le diagnostic de la grossesse estfait par échographie obstétricale. Toutes les patientes ont bénéficié d'un examen clinique complet biquotidien, un bilan biologique quotidien fait d'hémogramme, d'ionogramme, bilan hépatique, bilan rénale et de bilan d'hémostase. Le suivi gynécologique était hebdomadaire avec une échographie obstétricale et morphologique chez les patientes ayant reçues la chimiothérapie durant la grossesse. Le traitement, après consentement éclairé était différent selon le type de LA: selon le protocole LAL dans le cas des LAL et selon le protocole LAM dans le cas des LAM. Un bilan malformatif clinique et échographique a été fait chez tous les nouveau-nés.

## Résultats

Huit cas de LA au cours de la grossesse sont colligés au service d'hématologie du CHU de Casablanca. Sept leucémies aiguës myéloblastiques (LAM) et une leucémie aiguë lymphoblastique (LAL). Quatre cas sont diagnostiqués durant le 1er trimestre de la grossesse, 1 cas estdiagnostiqué pendant le 2ème trimestre et 3 cas pendant le 3ème trimestre. L'accouchement est par césarienne programmée dans 2 cas et par voie basse dans 3 cas dont un est prématuré, 2 fausses couches et une mort fœtal après décès maternel. Cinq patientes ont reçues la chimiothérapie durant la grossesse à des termes différents et avec des médicaments différents. Les 5 nouveau-nés sont bien portants avec un bilan malformatif négatif. Les caractéristiques des patientes, leurs traitements et évolutions figurent sur les Tableau 1 et Tableau 2.

**Tableau 1:** Les caractéristiques obstétricales, hématologiques et des Leucémies aigues des patientes

Cas	Age ans	Grossesse	Hémogrammee			Type de LA	Caryotyp	Age de grossesse (SA)
			Globules Blanc (éléments/mm3)	Hb (g/dl)	Plaquettes (éléments/mm3)			
**1**	23	Primipare	34 000	3	11 000	LAL b	Normal	32
**2**	27	Primipare	22 000	8	9000	LAM 4	Normal	33
**3**	21	Primipare	57 000	7	65 000	LAM 1	Normal	32
**4**	23	3G 3P	130 000	4	16 000	LAM2	Normal	21
**5**	20	Primipare	300 000	3	60 000	LAM2	T(8,21)	10
**6**	24	3G 3P	20 000	6	34 000	LAM2	Trisomie 8	17
**7**	19	2G 2P	280 000	5	20 000	LAM1	Non fait	13
**8**	34	5G 2P	30 000	6	8000	LAM1	Normal	24

G: nombre de gestation, P: nombre de parité, LAM: leucémie aigue myéloblastique SA: semaine d'aménorrhée, LAL: leucémie aigue lymphoblastique

**Tableau 2:** Traitement des patientes durant la grossesse et après l'accouchement ainsi que leurs devenirs hématologiques et obstétricaux

Cas	Age de grossesse en semaine d'aménorrhée	Traitement au cours de la grossesse	Devenir de la grossesse	Devenir fœtal	Traitement après accouchement	Statut de la mère
**1**	32	Prèdenisone: 60 mg/j pendant 4 j	Césarienne à 34 SA	Vivant, Bilan malformatif négatif	Chimiothérapie selon le protocole LAL	Mise en rémission complète (RC) après chimiothérapie d'induction décédé en phase de consolidation après rechute in thérapie
**2**	33	-	Accouchement prématuré par voie basse à 34SA	Vivant Bilan malformatif négatif	Chimiothérapie selon le protocole LAM	mise en RC après chimiothérapie d'induction décédé suite à une rechute après recul de 10 mois
**3**	32	Hydroxyurée: 3g/j pendant 4 j	Césarienne à 37 SA	Vivant Bilan malformatif négatif	Chimiothérapie selon le protocole LAM	Rémission complète maintenue (RCM) après un recul de 22 mois
**4**	21	-Hydroxyurée: 4 g/j pendant 21 j **- inductionI**: daunorubicine 80mg /j J1,J2,J3; Cytarabine 350mg/j pendant 7 j - **induction II:** daunorubicine 80mg /j pendant 3 j; Cytarabine: 350mg/j pendant 7 j	Accouchement par voie basse à 35 SA	Vivant Bilan malformatif négatif	Chimiothérapie selon le protocole LAM	Mise en RC après chimiothérapie d'induction RCM après un recul de 24 mois
**5**	10	- Hydroxyurée: 4g/j pendant 4 j - cytarabine 100 mg/j en perfusion de 1heure pendant 2jours	Fausse couche à 11 SA	Mort fœtal après chimiothérapie	Chimiothérapie selon le protocole des LAM	mise en RC après chimiothérapie d'induction décédé suite à une rechute après à un recul de 6 mois
**6**	17	**induction I**: daunorubicine 80mg /j pendant 3 j Cytarabine 300mg/j pendant 7 j **- induction II:** daunorubicine 80mg /j pendant 3 j Cytarabine 300mg/j pendant 7 j	Accouchement par voie basse à 37 SA	Vivant Bilan malformatif négatif	Chimiothérapie selon le protocole des LAM	mise en RC après chimiothérapie d'induction RCM avec un recul 3 mois
**7**	13	Flash de cytarabine à 100 mg/j pendant 2 j	Fausse couche à 14 SA	Mort fœtal après chimiothérapie	-	décédé au cours du traitement
**8**	24	Cytarabine: 300 mg/j pendant 4 j	Mort fœtale à 27 SA	Mort fœtal après décès maternel	-	Décédé au cours du traitement

G: nombre de gestation, P: nombre de parité, LAM: leucémie aigue myéloblastique SA: semaine d'aménorrhée, LAL: leucémie aigue lymphoblastique

## Discussion

Les hémopathies malignes associées à la grossesse sont rapportées dans la littérature: maladie d'hodgkin, lymphome non hodgkinien, LA, syndrome myélodysplasique [7, 8]. L'association leucémie et grossesse est rare, son incidence est estimée à 1 /75 000 – 100 000 [1, 2]. Le premier cas été publié par Virchow en 1845 [2]. Chez la femme enceinte 90% des leucémies sont aiguës avec 61% de LAM et 28% de LAL [9]. Le diagnostic est généralement fait pendant le 2ème et le 3ème trimestre de la grossesse [1, 9, 10]. Parfois le diagnostic est difficile du fait d'attribuer les symptômes de la LA à la grossesse [2]. Tout retard ou changement de traitement pour sauvetage fœtal risque d'augmenter le taux de mortalité maternelle. Il est admis que la grossesse n'affecte pas l’évolution ou le taux de rémission complète des LA [2].

La grossesse entraîne des changements métaboliques: l'augmentation d'environ 50% du volume plasmatique et de la clairance rénale, la création d'un troisième secteur par la cavité amniotique et l'accélération de l'oxydation hépatique [11, 12]. Ces modifications diminuent la concentration de la chimiothérapie chez l'animal. Cependant il n'y a pas d’étude sur la pharmacocinétique de ces médicaments chez la femme enceinte, ce qui renddifficile la détermination des doses chez ces patientes [11].

L'effet de la chimiothérapie chez le fœtus durant la période de l'organogenèse est caractérisé par une augmentation du taux d'avortement spontané, de mort fœtal 6%, de retard de croissance intra utérin 7%, de malformations fœtal et d'accouchement prématuré 5% [5, 13]. L'effet à long terme est mal connu. Après la période de l'organogenèse l'administration de la chimiothérapie est associée à un risque faible de retentissement sur le développement fœtal, l'hypotrophie reste l'incident prédominant [10, 14]. Certains auteurs considèrent que l'administration de la chimiothérapie pendant le 2ème et le 3ème trimestre est possible sans risque, cependant ceci n'est pas admis par d'autres auteurs [15–17].

Le traitement d'une LAM diffère de celui d'une LAL. Dansles LAM le protocole le plus utilisé consiste en l'association de cytarabine et antracyclines. L'expérience avec la cytarabine au cours de la grossesse est limitée. C'est un anti-métabolite connu par son effet tératogène selon les essaies clinique chez l'animal [18, 19]. Une revue de 93 cas de femmes enceintes ayant reçues durant la grossesse soit la cytarabine seule soit en association avec d'autres drogues (thioguanine, doxorubicine, prédenisone et vincristine) retrouve 4 cas de malformations rénales, 6 cas de mort fœtal in utero, 12 cas de retard de croissance intra utérin, 2 cas de mort néonatale liée à une infection sévère et 5 cas de cytopénie néonatal [11, 12].

Les antracyclines sont indispensables dans le traitement des LA. Ils sont cardiotoxiques pour le fœtus. L'idarubicine est proscrite durant la grossesse car elle a un passage placentaire important du fait de son caractère lipophile [20]. La doxorubicine semble être la plus utilisée, son usage est relativement sans risque [8, 12, 19, 21]. Une revue de 160 cas de femme enceinte exposées aux antracyclines retrouve2 cas de cardiopathie réversible liée à l'usage de l'idarubicine pendant le 2ème trimestre et un cas de cardiopathie létale [20]. Un suivi de 81 enfants issus de mères traitées par des antracyclines pendant la grossesse n'a pas retrouvé de cardiopathie ni pendant la grossesse ni pendant le post partum [19].

L’ Hydroxyurée est un médicament cytotoxique inhibiteur de la synthèse d'ADN. Elle doit être évitée pendant le 1er trimestre. Son usage durant le 2ème et le 3ème trimestre augmente le risque de survenue de pré- éclampsie. Une revue de 50 femmes enceintes traitées par Hydroxyurèe pour différentes indications retrouve 2 cas demort fœtal intra- utérine (traité pendant le 1er trimestre), 3 cas de malformations mineurs et 9 cas d'accouchement prématuré [21].

La leucémie promyélocytaire (LAM3) pose le problème de coagulopathie intra vasculaire disséminé (CIVD) qui risque d'entraver la prise en charge de la grossesse. Son traitement est basé sur l'acide tout transrétinoïque (ATRA) associé à la chimiothérapie. L'ATRA est tératogène dans environ 85%pendant le 1er trimestre de la grossesse [11].

L'administration de l'ATRA seul ou en association aux antracyclines pendant le 2ème et le 3ème trimestre est rapportée dans la littérature avec de bons résultats sans malformations associée [2, 20–25]. La prévalence des LAL au cours de la grossesse est rare, 19 cas sont rapportés dans la littérature [2]. C'est une maladie agressive nécessitant une chimiothérapie dès le diagnostic. Les données de la Littérature sont rares concernant le traitement des LAL, le méthotréxate représente un agent important dans le traitement mais il est connu par son effet tératogène. L'usage du mèthotrèxate pendant le 1er trimestre augmente le risque de fausse couche et la survenue de malformations: hypertélorisme, macrognathie et des anomalies de l'ouïe [26].

Les corticostéroïdes de courte durée d'action, Le prédnisone, le prédnisolone et le méthyl-prédenisolone sont métabolisés par la 11 hydroxgenase placentaire ce qui expose le fœtus à 10% de la dose maternelle [27]. Les études chez l'animal ont montré que l'exposition anténatale aux corticostéroïdes prédispose aux malformations de la fente palatine et au trouble du développement neuronal [27]. L'usage des corticostéroïdes chez la femme enceinte peut être associé à un retard de croissance intra-utérin avec hypotrophie [28].

Le risque de malformation diminue avec l'avancement de la grossesse, ainsi La décision thérapeutique doit prendre en considération l’âge de la grossesse et l'agressivité de LA. Au cours du 1er trimestre il est suggéré d'arrêter la grossesse puis le traitement. Au cours du 3ème trimestre le traitement est fait selon les protocoles standards. Au cours du 2ème trimestre l'interruption s'impose avant 20 SA et après 20 SA le traitement doit prendre en considération le retentissement des drogues chez le fœtus [15, 28].

## Conclusion

L'association d'une LA et grossesse est un évènement rare. Elle nécessite une prise en charge multidisciplinaire tenant compte des impératifs de la maladie et de son traitement, de la femme et de son désire de grossesse ainsi que des dimensions éthiques et morales. L'utilisation de chimiothérapie au cours de la grossesse est possible après 20 SA. L'interruption de grossesse s'impose avant cette date. Les résultats des traitements des LA pendant la grossesse se comparent favorablement à ceux obtenus dans une autre situation, à intensité égale de traitement. Il persiste des doutes sur l'avenir des enfants issus de ces grossesses compte tenu de la faible quantité de données, ce qui justifierait la création d'un registre pour un suivi à long terme qui permettrait de proposer des thérapeutiques Plus adaptées.

## References

[1] Tae Sung Park, Seung Tae Lee, Jin Seok Kim (2009). Acute promyelocytic leukemia in early pregnancy with translocation t(15;17) and variant PML/RARA fusion transcripts. Cancer Genetics and Cytogenetics..

[2] Chelghoum Y, Vey N, Raffoux E (2005). Acute leukemia during pregnancy: a report on 37 patients and a review of the literature. Cancer..

[3] Avilés A, Neri N (2001). Hematological malignancies and pregnancy: a final report of 84 children who received chemtherapy in utero. Clin Lymphoma..

[4] Pereg D, Koren G, Lishner M (2008). Cancer in pregnancy gaps challenges and solutions. Cancer Treat Rev..

[5] Pacifici GM, Nottoli R (1995). Placental transfer of drugs administered to the mother. Clin Pharmacokinet..

[6] Dilek I, Topcu N, Demir C (2006). Hematological malignancy and pregnancy: a single-institution experience of 21 cases. Clin Lab Haematol..

[7] Anatolian Medical Oncology Society Group (2010). Malignancies diagnosed during pregnancy and treated with chemotherapy or other modalities (review of 27 cases): multicenter experiences. Int J Gynecol Cancer.

[8] Hurley TJ, McKinnell JV, Irani MS (2005). Hematologic malignancies in pregnancy. Obstet Gynecol Clin North Am..

[9] Ali R, Ozkalemkas F (2009). Acute leukemia and pregnancy. Leuk Res..

[10] Shapira T, Pereg D, Lishner M (2008). How I treat acute and chronic leukemia in Pregnancy. Blood Rev..

[11] Weisz B, Meirow D, Schiff E, Lishner M (2004). Impact and treatment of cancer during pregnancy. Expert Rev Anticancer Ther..

[12] Cardonick E, Iacobucci A (2004). Use of chemotherapy during human pregnancy. Lancet Oncol..

[13] Tashiro H, Umezawa K, Shirota M, Oka Y, Shirasaki R, Nishi R, Taguchi A, Akiyama N, Kawasugi K, Ayabe T, Shirafuji N (2011). Acute myelogenous leukemia developed at the 26th week of gestation. Rinsho Ketsueki..

[14] Abadi U, Koren G, Lishner M (2011). Leukemia and lymphoma in pregnancy. Hematol Oncol Clin North Am..

[15] Niedermeier DM, Frei-Lahr DA, Hall PD (2005). Treatment of acute myeloid leukemia during the second and third trimesters of pregnancy. Pharmacotherapy..

[16] Papantoniou N, Daskalakis G (2008). Management of pregnancy in adolescence complicated by acute lymphoblastic leukemia. Fetal Diagn Ther..

[17] Germann N, Goffinet F, Goldwasser F (2004). Anthracyclines during pregnancy: embryo-fetal outcome in 160 patients. Ann Oncol..

[18] Avilés A, Neri N, Nambo MJ (2006). Long term evaluation of cardiac function in children who received anthracyclines during pregnancy. Ann Oncol..

[19] Siu BL, Alonzo MR (2002). Transient dilated cardiomyopathy in a newborn exposed to idarubicin and all-trans-retinoic acid (ATRA) early in the second trimester of pregnancy. Int J Gynecol Cancer..

[20] Pereg D, Koren G, Lishner M (2007). The treatment of Hodgkin's and non-Hodgkin's lymphoma in pregnancy. Haematologica..

[21] Thauvin-Robinet C, Maingueneau C (2001). Exposure to hydroxyurea during pregnancy a case series Leukemia..

[22] Breccia M, Cimino G (2002). AIDA treatment for high-risk acute promyelocytic leukemia in a pregnant woman at 21 weeks of gestation. Haematologica..

[23] Delgado-Lamas JL, Garcés-Ruiz OM (2000). Malignancy: Case Report: Acute Promyelocytic Leukemia in Late Pregnancy. Successful Treatment with All-Trans-Retinoic Acid (ATRA) and Chemotherapy. Hematology..

[24] Azim HA, Peccatori FA, Pavlidis N (2010). Treatment of the pregnant mother with cancer: a systematic review on the use of cytotoxic, endocrine, targeted agents and immunotherapy during pregnancy. Part I: Solid tumors. Cancer Treat Rev..

[25] Addar MH (2004). Methotrexate embryopathy in a surviving intrauterine fetus after presumed diagnosis of ectopic pregnancy: case report. J Obstet Gynaecol Can..

[26] Blanford AT, Murphy BE (1977). In vitro metabolism of prednisolone, dexamethasone, betamethasone, and cortisol by the human placenta. Am J Obstet Gynecol..

[27] van Runnard Heimel PJ, Franx A (2005). Corticosteroids, pregnancy, and HELLP syndrome: a review. Obstet Gynecol Surv..

[28] Pentheroudakis G, Pavlidis N (2006). Cancer and pregnancy: poena magna, not anymore. Eur J Cancer..

